# On the Dynamics of Transient Plasmas Generated by Nanosecond Laser Ablation of Several Metals

**DOI:** 10.3390/ma14237336

**Published:** 2021-11-30

**Authors:** Stefan Andrei Irimiciuc, Sergii Chertopalov, Michal Novotný, Valentin Craciun, Jan Lancok

**Affiliations:** 1 National Institute for Laser, Plasma and Radiation Physics—NILPRP, 409 Atomistilor Street, 077125 Magurele, Romania; valentin.craciun@inflpr.ro; 2 Institute of Physics of the Czech Academy of Sciences, Na Slovance 2, 182 00 Prague, Czech Republic; novotnym@fzu.cz (M.N.); lancok@fzu.cz (J.L.); 3 Extreme Light Infrastructure for Nuclear Physics, IFIN-HH, 077125 Magurele, Romania

**Keywords:** laser produced plasmas, metals, Langmuir probe, plasma-target correlation

## Abstract

The dynamics of transient plasma generated by UV ns-laser ablation of selected metals (Co, Cu, Ag, Bi) were investigated by the Langmuir Probe method in angle- and time-resolved modes. Multiple ionic and electronic structures were seen for all plasmas with some corresponding to anions or nanoparticle-dominated structures. The addition of an Ar atmosphere energetically confined the plasma and increased the charge density by several orders of magnitude. For pressure ranges exceeding 0.5 Pa fast ions were generated in the plasma as a result of Ar ionization and acceleration in the double layer defining the front of the plasma plume. Several correlations between the target nature plasma properties were attempted. The individual plasma structure expansion velocity increases with the melting point and decreases with the atomic mass while the corresponding charged particle densities decrease with the melting point, evidencing the relationship between the volatility of the sample and the overall abated mass.

## 1. Introduction

Laser ablation of metals has been the focus of several studies in the past 30 years. The outcome of those studies has impacted both the fundamental knowledge of the laser ablation process and important applications like pulsed laser deposition [1,2], nanoparticle generation [3,4], laser patterning [5], or compositional analysis [6,7]. Several theoretical routes were developed to model the laser ablation of metals. Zighilei et al. [8,9], developed a model based on atomistic modeling considering phenomena like melting, spallation and phase explosion while Bykov et al. [10] developed a hybrid model based on integrating the thermal problem with Monte Carlo simulation and later updated the model for the ultrashort laser ablation of Cu, Ag and W. Luney et al. [11] developed a model that considers the vaporization of the metals and post ablation ionization, while Wood et al. [12] developed a multiple scattering hydrodynamic model to describe the dynamics of ablation plasmas expanding in a background gas. Although the theoretical modeling sometimes can be aimed at understanding the fundamentals of the ablation process exclusively, there are some experimental studies attempting to find a correlation between plasma composition and dynamics and the irradiated targets. Baraldi et al. [13] investigated the dynamics of metallic ions (Au, Cu, Ag, Al, Bi) generated by UV ns laser ablation at fluences up to 15 J/cm^2^ and found that the ablated yield decreases with the melting temperature (or cohesive energy) of the metals. Similar dependence on the cohesive energy was reported by Therstrup et al. [14] and Salle et al. [15] which used alternative approaches to assess the ablation efficiency in UV-ns laser ablation of metals as functions of the ablation yield (ablated mass) of the cohesive energy (melting point). Konomi et al. [16] investigated the angular distribution dependence of the plasma properties in the case of metallic targets. The authors concluded that the low atomic mass elements have a wider angular distribution, results confirmed in [17] when the ablation of a complex multi-element target was investigated. In recent years Anoop et al. [18,19,20] and our group [21,22,23,24,25,26] used extensive diagnostic investigations on metallic plasma and revealed clear dependence of some plasma parameters on the melting point, atomic mass, or electrical conductivity of the material. The reports are general and cover several irradiation regimes (fs, ps, ns) and a wide range of materials (Al, Cu, Mn, Ni, In, Te, W, Zn, Ti). 

A significant part of available results on the target-plasma relation is given through the implementation of the Langmuir probe method in its time-resolved [24] or collector [27] versions. We recently showed that for the case of pulsed laser deposition (PLD) in specific conditions the information extracted from the saturation current of the probe can be limited without the implementation of complementary, usually more expensive techniques (high resolution optical emission spectroscopy, mass spectrometry, etc.). In [24,25,28] we proposed the time and angle-resolved analysis of unbiased Langmuir Probe (LP) as an alternative to the usual approach, which has the advantage of offering more precise information relating to the internal structure of the ionic current, which is not possible in the saturation regime and gives the possibility to study both positive and negative charges from the ablated plasma. Recently, the method was shown to be sensitive to Ag oxidation processes in the plasma volume for pressure as low as 0.5 Pa. 

This paper used the LP for angle- and time-resolved analysis to investigate common trends in transient plasmas generated by ns-laser ablation of metals. The charge velocity distribution of plasma along various expansion angles was used to investigate the ion and electron distribution. The effect of Ar gas on the dynamics of metallic plasma was also studied and specific thresholds of gas ionization were found. By using a semi-empirical model, we calculated the neutral plasma density and plasma core temperature and we correlated them with the melting point and atomic mass of the targets. Other several target-plasma correlations were also found for electron temperature, charge density and expansion velocity. 

## 2. Materials and Methods

**Figure 1:**
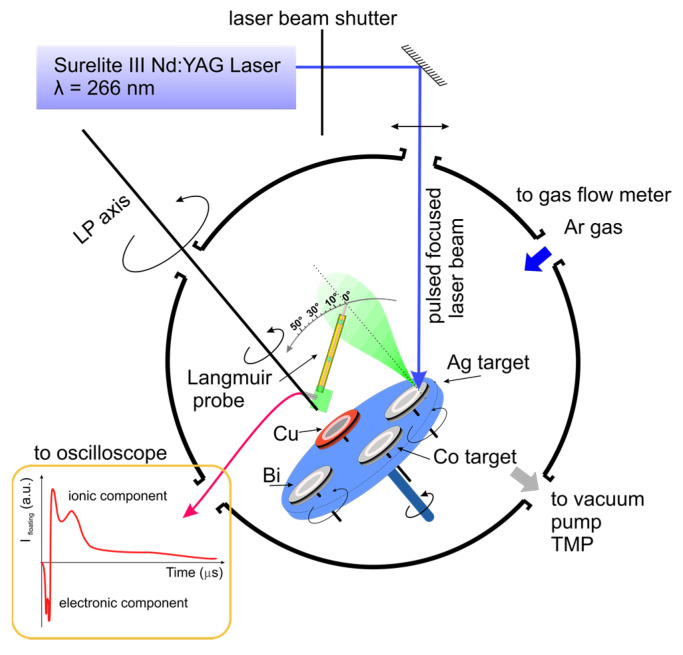
Experimental Set-up.

A Surelite III Nd:YAG laser (wavelength—266 nm, repetition rate—10 Hz, fluence—3.8 J/cm) was focused on several metallic targets (Co, Cu, Ag, Bi). The targets were continuously rotated to avoid local heating and deep crater formation. The target-to-substrate and LP distances were 50 mm and 37 mm, respectively. The plasma investigations were performed under a residual vacuum of 5 × 10^–5^ Pa and at 5 × 10^–2^, 5 × 10^–1^, 2, 5, and 10 Pa Ar pressure. Each experiment was preceded by a target surface cleaning procedure during which the LP was shielded from the incoming transient plasma while the rotating target was laser irradiated with 1200 pulses (for other details on the setup geometry, see Figure 1). The signal from the tungsten LP (diameter—0.2 mm and exposed length—2 mm) was measured by collecting the voltage signal across a load resistor with a Tektronix DPO 4104 Digital Phosphor Oscilloscope (1 GHz, 5 GS/s, Tektronix Inc. Beaverton, OR, USA). All performed investigations were time-synchronized by a fast silicon photodiode (Thorlabs FDS100, Thorlab, Dortmund, Germany), and the initial measuring moment was considered to be the moment when the laser was fired.

## 3. Results and Discussions

### Unbiased Probe Investigations of Metallic Plasmas

**Figure 2:**
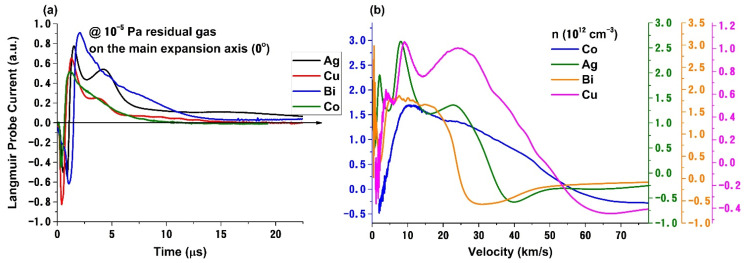
Un-biased probe current for several metallic plasmas (**a**) and the corresponding charge density distribution with expansion velocities (**b**).

In Figure 2a we have represented the unbiased probe signals recorded at 0° (along the main target normal axis) of each investigated plasma in a free expansion regime. We observe that all the temporal traces have similar features: an electronic component for arrival times below 1–2 μs and an ionic contribution for longer arrival times with specific characteristics to each material. The resulted ions can be directly ablated from the target, induced by single or multi-photon ionization processes, or generated through collisions in the early stages of ablation or by the plasma-working gas interactions. The dominant mechanisms are often dictated by the laser wavelength and fluence, target nature, or background gas pressure. Plasma generated in a vacuum on Ag or Cu has a clear three-peak structure (1st structure: Ag—22 km/s, Cu—28 km/s; 2nd structure Ag—8 km/s, Cu—12 km/s, 3rd structure Ag—2 km/s, Cu—4 km/s). Each peak, according to our previous results [25] and from the model proposed by N. Bulgakova’s group [29,30], corresponds to a different ionization state. For the case of Cu, the three-peak structure in the ion density distribution has also been reported by S. Harilal’s group [31] at higher laser fluences, with the difference in fluence being explained by the use of saturation current as the main investigation instrument. For the Co and Bi, the plasma structuring is not obvious. This change can be interpreted to be induced by the differences in the properties of the irradiated target. Based on our previous work from [32] we showed that in the fs, ps and ns regimes the acceleration field can be correlated with the electrical conductivity of the target through the density of available electrons to be removed during the ablation process. Therefore, we can extend the same paradigm here and correlate the degree of structuring with the target’s electrical conductivity as the strengths of the acceleration potential will be reflected in the expansion velocities of ionized species [26,29]. For all investigated plasma the electronic and ionic peaks shift towards lower arrival times as the atomic mass of the irradiated target decreases. This result confirms the interpretation of the LP temporal traces with a Coulomb-shifted Maxwell Boltzmann distribution where different contributions to the particle velocity (thermal, Coulomb and adiabatic) have a $\sqrt{1/m}$ type contribution [33]. 

Using the procedure from [21] we reconstructed the charge density as a function of particle expansion velocity. We observe that in a high vacuum the structuring of the plasma is well in line with the previous data. Bi plasma is characterized by a narrow range of velocities of the ion structures (1st structure—21 km/s, 2nd structure—8 km/s, 3rd structure—1.5 km/s) and a dominant peak around 1–2 km/s which, according to the work of [4,34,35] can be correlated with a nanoparticle (NP) dominated structure. We have confirmed the nature of this slow structure in a recent paper [24] where we investigated the deposition of Ag nanoparticles by PLD. The widest distribution with ions having velocities up to 60 km/s is the Co plasma defined by low atomic mass particles which can gain more kinetic energy during the acceleration in the initial stages of ablation. 

With such complex distributions seen for all investigated plasmas with multiple maxima and possible NP-induced structures, the control of plasma energy during the deposition becomes essential. Several routes for controlling the plasma kinetics are proposed in the literature including but not limited to fluence reduction [2,36], background gas addition [22,37,38], off-axis deposition [39], etc. The control from laser fluence can be challenging as changes in the transferred energy towards the target strongly affects the fundamental ablation mechanisms and often leads to the formation of clusters and droplets on the deposited film [40,41], which is undesirable. Therefore, in the following, we will only focus on the latter two approaches. The addition of Ar as an inert gas up to 10 Pa will lead to an energy transfer from the plasma plume towards the gas particles via collisions. 

**Figure 3:**
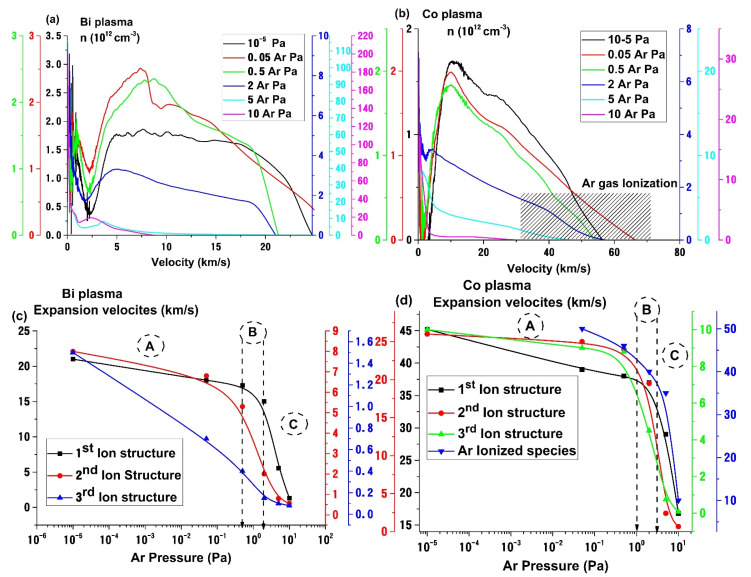
Pressure effect on Bi (**a**) and Co (**b**) ion velocity distribution and the deconvolution of the Bi (**c**) and Co (**d**) distribution characterizing the plasma expansion at 10^−5^ Pa.

In Figure 3a,b we plotted the ion particle velocity distributions at various Ar gas pressures. For the case of Cu plasma, the wide distribution seen in high vacuum conditions is confined towards lower velocity ranges from 28 km/s for the first structure to 3 km/s, from 12 km/s for the second structure to 1.3 km/s and from 4 km/s for the third structure to 0.2 km/s. For the case of Ag plasma, the plasma velocities extracted from charged particle distribution vary from 24 km/s for the 1st structure to 2 km/s, from 11.8 km/s for the 2nd structure to 3.5 km/s and from 3 km/s for the 3rd structure to 0.15 km/s. For the case of Bi, we can see variations from 21 km/s down to 1.3 km/s for the 1st structure, for the 2nd one from 8 km/s to 0.7 km/s and lastly for the 3rd one from 1.5 km/s to 0.08 km/s. Finally, for the case of Co plasma, the particle velocity distribution seen in high vacuum conditions is confined from 42 km/s for the first structure to 16.8 km/s, from 25 km/s for the second structure to 0.66 km/s and from 10 km/s for the third structure to 0.14 km/s. These values are roughly in line with the results from [42] where are reported usual ranges for particle velocities for higher laser fluence conditions. The group of Anoop et al. [43] using a wide range of fluences and a shorter pulse width reported tens of km/s for the ions and the front of the plasma and a few km/s for the slower plasma structure dominated by atoms. Mahoney et al. [44] under similar conditions of laser wavelength and fluence reported velocities of 10–20 km/s for the expanding plasma with a considerable reduction when adding O_2_ in the chamber.

In Figure 3c,d we have represented the Ar pressure effect on the expansion velocity of each structure. For the case of Bi, the three-structure seen in high vacuum evolves into a two-structure configuration (from 0.05 Pa) with the two ionic groups with the slowest velocities merging together. For the case of Co (presented here) and for Ag and Cu (not shown) we observe all the three expansion regimes. The *A* region characterizes the free expansion regime where only collisions occur within the plasma volume and the expansion velocities suffer a decrease of approximately 10% up to 0.5 Pa. This pressure value represents the threshold (*B*) which signals the transition from free expansion towards a collision-driven regime (*C*), for which we see a steep drop of 30%. The velocities attributed to the ionized Ar plasma (blue curve in Figure 3d) are of tens of km/s range and close to the values of the fastest ion group which confirms their origin. The ionized Ar is generated only at the front (edges) of the plume where the electrons and fast ions have enough energy to ionize and excite the Ar gas atoms. For many metallic plasmas (Cu, Ag, Co) the most probable velocities of the electrons are in the hundreds of km/s range. The resulting kinetic energies for the front of the plasma would be around 500 eV in the case of Co, 270 eV for Cu, to 260 eV for Ag and 200 eV for Bi. These values considerably surpass the ionization potential of Ar (15.3 eV). From 0.5 Pa Ar pressure the mean free path decreases from 1.3 cm to 6.5 × 10^−2^ cm at 10 Pa, thus the appearance of a new peak is correlated with Ar gas ionization during expansion. For the case of Bi, the ejected charge energy coupled with the wider angular distribution led to fewer Ar gas ionization, as no new peak was detected in Bi particle velocity distribution. 

We recently showed by using optical emission spectroscopy that Ag plasma can ionize Ar gas during the deposition of Ag NP and influence the structural quality and surface morphology of the films. Regarding the background gas, Isaac et al. [45] have even reported the full ionization of 27 Pa of Ar gas in the vicinity (0.6–1 mm) of the Ag target by means of OES investigations while Amoruso et al. [46], when investigating the laser ablation of MgB_2_ in 10 Pa of Ar gas reported similar conclusions.

**Figure 4:**
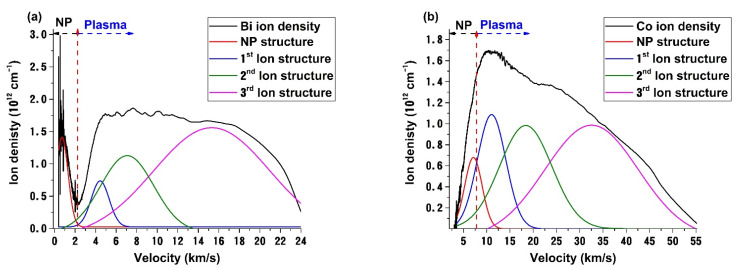
Deconvolution of the charge density velocity distribution of Bi (**a**) and Co (**b**) plasmas for the 10^−5^ Pa case.

To better understand the charged particle distributions, we performed a deconvolution of the measured traces. Selected data on Co and Bi plasma expanding in 10^−5^ Pa are presented in Figure 4a,b. The general laws describing the particle distribution in laser produced plasmas are defined, as reported in [47,48], through Coulomb shifted Maxwell Boltzmann distributions. The fitting procedure follows the separation between the plasma structures, with three peaks characterized by high velocities and the NP structure, which is defined by a low expansion velocity. Our analysis also showed that the NP structure has the largest FWHM compared to any other plasma structure. In the case of Bi, we found 5.9 km/s for NP while for the three plasma structures we found Ag—5 km/s, Cu—4.5 km/s and Co—4 km/s, respectively. The presence of high energetic groups of ions within the plasma when relating the data to pulsed laser deposition raises the question of re-sputtering of the deposited film. For the second and third structures in vacuum conditions, the kinetic energies of the investigated plasmas are Co 2nd—185 eV, Cu 2nd—50 eV. Ag 2nd—35 eV. Bi 2nd—16 eV. and Co 3rd—185 eV, Cu 3rd—50 eV. Ag 3rd—35 eV. Bi 3rd—16 eV. This means that there is a strong possibility in vacuum conditions for re-sputtering to be a key phenomenon during the deposition process, as the bonding energy of the deposited metals are considerably lower (Ag: 1.68 eV; Co: 1.31 eV; Cu: 2.08 eV; Bi: 2.11 eV). The situation changes from 0.5 Pa upwards, when the kinetic energies are decreasing enough to avoid the appearance of re-sputtering; under these conditions, the deposition/growth rate of the film should increase significantly. This is confirmed experimentally as it was previously reported in [44,49], but there wasn’t given a quantifiable set of data that can be transferable to other deposition conditions as is given here. 

**Figure 5:**
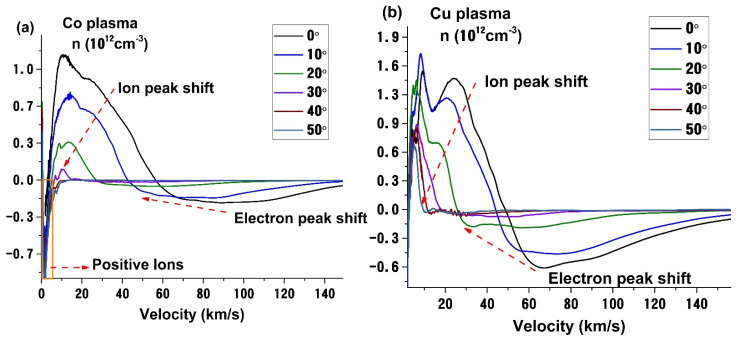

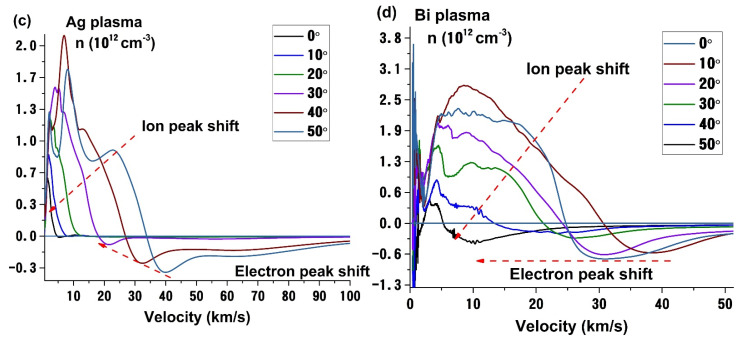
Angular dependence of charged particle velocity distribution for the investigated metallic plasmas (Co (**a**); Cu (**b**); Ag (**c**); Bi (**d**)).

As laser produced plasmas present a strong angular distribution of their properties it is important to see how these properties can potentially affect the deposition process. For all the investigated metals and based on the previously available data, most plasmas have a similar angular distribution. The general evolution follows a cos^n^ (θ) type function, which was shown for a wide range of the particle-based on saturation current measurements and thin quartz crystal microbalance or thin-film thickness measurements [50,51]. In one of our previous works, we showed, based on their energy, that not all plasma species follow the same angular trend, with low energy particles presenting a strong shoulder towards higher angles. The latter was found here for Bi plasma which has particles with higher kinetic energies towards the edges of the plasma, as opposed to Co, Ag, and Cu, which have an energetic maximum in the center of the plasma plume. Similar results were reported in [17,52] where a strong angular heterogeneity based on the mass of the ablated particles seen through optical emission spectroscopy and mass spectrometry measurements was observed. From the selected metals investigated here (seen in Figure 5), Ag and Cu plasmas have a strong angular gradient with velocities decreasing from 24 km/s at 0° to 2 km/s at 50° for Ag and 28 km/s at 0° to 8 km/s at 50° for Cu. The shift of the distribution towards low energies is seen also for electron-dominated structures which are accompanied by a steep decrease in electron density of 90% for Ag and 42% for Cu. A special case is seen for Co plasma where a negative peak in the distribution is characterizing low energy particles. Based on their velocity and the data reported in [52], the peak should correspond to positively charged particles. The position of the maximum does not vary, remaining centered around 2 km/s, however, its amplitude increases with about one order of magnitude from 3.4 × 10^11^ to 3.4 × 10^12^ when the measuring angle is varied from 0° to 50°. Based on this behavior we can conclude that this slow structure is not affected by the initial acceleration of the ejected charges, signaling a longer incubation time for their ejection. The variation in intensity would be correlated with the energy distribution of the laser beam at the irradiated surface and suggests a thermal mechanism for ejection (explosive boiling or thermal evaporation). Similar behavior is also seen for Bi, where the initial peak identified as NP in the previous section, changes its charge towards the edge of the plasma. Additionally, the area highlighted in Figure 4d represents a secondary contribution of the positive Bi ions for wide measuring angles from 30° to 50°. The presence of metal anions in laser-produced plasmas has only been reported by the use of time-of-flight mass spectrometry measurements [53,54,55,56]; this is one of the first attempts to extend the LP probe technique towards understanding positive ion dynamics during the deposition process. 

## 4. Target Properties Influence on Plasma Dynamics

**Figure 6:**
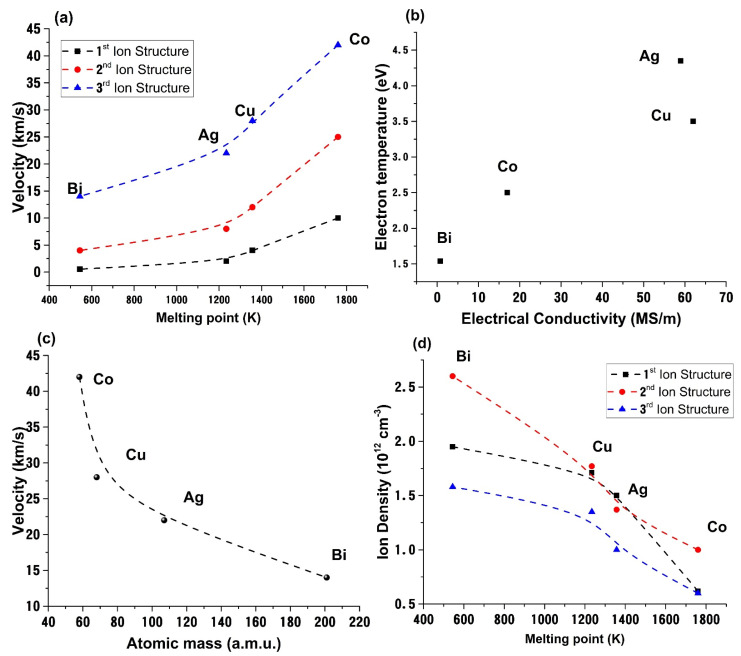
Dependence of expansion velocity on melting point (**a**), electron temperature on target electrical conductivity (**b**) expansion velocity on atomic mass (**c**) and ion density on melting point (**d**); lines are guide to the eye.

Finally, let us focus on the possible relation between the properties of the targets and the ones of the plasma. Our approach only considers the unbiased probe current as a true measure of the charge density dynamics. This puts us in an advantageous position as we can analyze in one step various ionic and electronic plasma structures, including, as we have shown, NP and positive ions. Some relations were found between the melting temperature, atomic mass, target’s electric conductivity and plasma parameters like expansion velocity, ion density, or electron temperature. The data are presented in Figure 6. We observe that the velocities of the ejected ions have a clear dependence on the atomic mass, thus confirming the nature of the measured velocity is of Coulomb nature and given by the relation [57] $\sqrt{\frac{2zeV_{0}}{m}}$, with *z*—the charge state, *e*—the electron charge and $V_{0}$—the accelerated voltage formed at the edges of the double layer formed during expansion. 

Additionally, the velocity is seen to have a quasi-exponential increase as a function of the melting temperature. With the increase of the melting temperature the vaporization and overall thermal ablation mechanism are hindered with the laser energy being transferred towards electrostatic ablation and charged particle acceleration. The electron temperature, determined using the procedure from [21], increases with the increase of target conductivity, which confirms the results from [26] where different sets of metals were investigated. The temperature increases as a result of the change in the electrostatic/thermal mechanism balance occurring in the initial stages of ablation which leads to an enhancing of the electron *thermal* movement within the plasma plume. Although, here we don’t have the same function describing the σ (T_e_) function as previously reported on [32] we still find a proportionality between the two parameters. This is explained by the larger measurement distance used here and the smaller range of investigated materials. Finally, when representing the charged particle density as a function of the melting point of the investigated materials we found that they follow a decreasing trend. The latter dependence is sustained by reports [14,15] for ns laser produce plasma and confirmed also for shorter pulse lengths [58] where the decrease in target volatility defined by an increase of cohesion energy or melting temperature of the target will lead to the ejection of a lower particle density. These results concerning the plasma properties and their angular distribution for the selected metals will potentially impact the efficiency of metal nanoparticles production either for plasmonic or magnetic application for effective fabrication of magnetoplasmonic structures [59,60].

Although LP is limited to the measurement of charge structure from the plasma (particle, clusters, NP, etc.) by implementing a semi-empirical model [47] on the time-of-flight data we can extract a series of parameters that characterize global parameters of the plasma-like, neutral temperature, average ionization state or acceleration field. Our data shows that the neutral densities generated through the ablation mechanism are lower than the ionic ones. The highest neutral density was found for Co: 6.1 × 10^10^ cm^−3^ followed by Ag: 3.1 × 10^10^ cm^−3^ and Cu: 1.6 × 10^10^ cm^−3^ with the lowest values obtained for Bi: 2 × 10^8^ cm^−2^. This result contradicts the LP data which shows higher densities being correlated with the melting point of the sample. To understand this difference, it is important to take into account the NP formation and/or direct ejection from the sample and to consider the difference of about 2 orders of magnitude between Ag, Cu, Co and Bi. These calculations show that although the plasma respects the dependence of the target’s volatility, the neutral core of the plasma could lose an important percentage towards NP or clusters formation, results confirmed from angle resolved LP where we saw important NP contribution of Bi as opposed to Ag or Cu. The average ionization states were calculated and we found that Cu (4) and Ag (3.5) have higher ionization states, while Bi (0.9) and Co (2) were on the other end of the distribution. We noticed that these differences are well correlated with the differences seen in the acceleration field (ex: Cu: 4.6 MV/cm and Bi: 0.5 MV/cm) and are causing the difference in expansion velocities between the investigated plasmas and the structuring of the charged particle velocity distribution. 

## 5. Conclusions

Extensive investigations based on angle and time-resolved unbiased Langmuir probe measurements were performed on a series of metals relevant to nanoparticles production via pulsed laser deposition. The measurements on the main expansion axis revealed a complex structuring of the electronic and ionic contributions to the probe current. The degree of plasma structuring was correlated with the average ionization states and the acceleration field generated in the incipient moments of ablation. The addition of Ar gas led to the confinement of the plasma towards lower expansion velocities and the increase by several orders of magnitude in ion density. Above 0.5 Pa Ar pressure, the generation of a new, faster ionic structure is seen and was attributed to the ionization of Ar gas during expansion. The balance between the energy of the plasma structure and the Ar ionization process sustains the proposed hypothesis. For the case of softer materials (Bi), we saw that a supplementary plasma structure, attributed to the formation of metallic nanoparticles from the gas phase, is visible from 10^−5^ Pa. The formation of a higher density of NP was reflected in the drop of neutral particle density by several orders of magnitude. Several correlations were made between the target -plasma properties. The expansion velocity was seen increasing with the increase of melting points and decreasing with the atomic mass, revealing a dominance of the electrostatic mechanism in charged particles acceleration. The charged particles density was found to decrease with the increase of the melting point, anchoring the relationship between the volatility of the sample and the overall abated mass.

## Data Availability

The data that support the findings of this study are available from the corresponding author upon reasonable request.
